# How Does Digitalization Affect Haze Pollution? The Mediating Role of Energy Consumption

**DOI:** 10.3390/ijerph191811204

**Published:** 2022-09-06

**Authors:** Jing Wang, Yubing Xu

**Affiliations:** College of Economics and Management, Northeast Agricultural University, Harbin 150030, China

**Keywords:** digitalization, energy consumption, haze pollution, mediating effect

## Abstract

In the context of digital technology innovation, an in-depth investigation into the impact of digitalization on haze pollution is of great significance for scientifically understanding environmental effects of digitalization and building a livable civic environment. From the perspective of energy consumption intensity and structure, this paper theoretically analyzes the direct and indirect effects of digitalization on haze pollution. On this basis, the impact of digitalization on haze pollution for 81 countries over the period 2010–2019 is empirically investigated by using the system GMM and mediating effects model. Empirical results show that digitalization can effectively suppress haze pollution, and there is significant heterogeneity in this inhibiting effect. In addition, digitalization can indirectly restrain haze pollution by reducing energy consumption intensity and optimizing energy consumption structure. The findings of this paper can provide enlightenment for countries to promote digitalization, combat haze pollution, and thus enhance the health of community residents.

## 1. Introduction

In meteorology, fog refers to a large number of tiny water droplets floating in the air, which makes horizontal visibility less than 1.0 km; smog refers to fine dry particles floating in the air, which makes visibility less than 10.0 km. A mixture of the two is commonly referred to as “haze” [1]. The main component of haze is ${\mathrm{PM}}_{2.5}$, which can carry harmful substances into the lungs and the bloodstream due to its small diameter and large contact area [2,3]. This not only directly threatens the mental and physical health of mankind, but also indirectly causes huge economic losses [4]. After testing the air quality of 6000 cities in 117 countries worldwide, the World Health Organization found that almost 99% of the world’s population is exposed to haze pollution. According to the global real-time air quality index report, haze pollution has become the most serious environmental problem that plagues and threatens physical fitness in the contemporary world [5]. Obviously, the grim situation of haze pollution no longer allows governments to passively wait for the inflection point of the Kuznets Curve (EKC) to appear automatically. Under this background, how to effectively control haze pollution has become the main focus of governments and academia [6].

Since the beginning of the 21st century, the global economic system has undergone tremendous changes with the rapid development and continuous penetration of information and communication technologies such as the mobile internet, internet of things, big data, cloud computing, and blockchain. According to the International Data Corporation (IDC), the total amount of global data will grow from 33 ZB in 2018 to 175 ZB in 2025 [7]. Human beings are going through the process of “digitalization” from traditional social forms to digital social forms. Different from the simple application of general ICT, digitalization encompasses various possibilities offered by the implementation of general ICT, which can range from the use of basic technologies (the application of computers or the internet) and modern technologies (the application of automation, cloud computing, and big data) to progressive applications (the adoption of business models or production processes based on digital technology products and services) [8]. In the process of digitalization, the deep embedding of massive data resources and digital technologies in the fields of energy, resources and the environment, especially in key fields such as power, industry, transportation, and construction, has injected new momentum into promoting economic transformation and development [9]. Then, there is an urgent question to be answered: can digitalization curb haze pollution? If this hypothesis is logical, how does digitalization suppress haze pollution, and what is its functional mechanism? Specifying these problems can provide a theoretical basis and empirical support for countries to combat haze pollution and achieve sustainable development goals.

Regarding haze pollution, academics have conducted a lot of research into the investigation of the formation of haze pollution [10,11,12,13] and the main measures for its elimination [14,15,16]. Haze is a form of air pollution, and its formation is relatively complex, with objective natural causes and subjective artificial causes [13]. In the objective natural environment, climate, vegetation, terrain [12], low wind speed, and high relative humidity can trigger haze pollution [11]. In subjective human activities, burning coal for heating in winter, traffic, and industrial production are considered the main causes of haze pollution [10]. Although haze is essentially a natural phenomenon, influenced to some extent by the natural environment, in the final analysis it is caused by inappropriate human economic activity. Therefore, some scholars have adopted the general equilibrium (CGE) model to conduct in-depth research on economic levers for haze pollution control [14,15]. The most frequently used economic means for haze pollution control is taxation, which can be divided into sulfur tax, resource tax, and carbon tax depending on the object of the levy [16,17,18].

In terms of digitalization, many of the scholars focus their research on the economic effects of digitalization. For example, some scholars believe digitalization can improve labor productivity [19], drive industrial structure upgrades [20], narrow the income gap [21], and shape urban spatial structures [22] by optimizing the allocation of factor resources. However, there is sparse literature directly concentrating on the relationship between digitalization and haze pollution. Most studies tend to explore the logical relationship between information and communication technology (ICT) and carbon emissions, and there is no consensus on whether ICT can effectively reduce carbon emissions. Some scholars hold that the development and application of ICT is not only conducive to promoting the formation of green and low-carbon lifestyles of residents [23], but also empowers enterprises to intelligent green manufacturing and energy management, leads green public welfare and service innovation, achieves double improvement of production efficiency and carbon efficiency, and gradually becomes a powerful driving force for low-carbon transformation [24]. However, other scholars argue that digitalization brings not only the opportunities for haze pollution control but also great challenges [25]. In the initial stage of digitalization, the development and extensive use of ICT-related products has led to increased energy use and environmental pollution [26]. According to statistics, the energy consumption caused by the adoption of ICT products has increased rapidly at an annual rate of 7% in recent years [27]. By 2012, the global energy consumption caused by the use of ICT-related products has risen to 4.7%, which has increased by 3.9% compared to 2007 [28].

In summary, scholars have carried out a large number of studies on digitalization and environmental pollution, which provide valuable references for this paper to explore the logical relationship between digitalization and haze pollution. However, most existing studies focus on exploring the economic welfare and direct environmental effects of digitalization in local areas. There is only sparse literature that analyzes how digitalization takes its multiplier effect on optimizing resource allocation and thus suppressing haze pollution from a global perspective. Based on this, the marginal contribution and potential value of this paper are mainly reflected in the following aspects: First, this study breaks through the perspective of previous research that focuses solely on specific regions and incorporates digitalization into the framework of haze pollution control based on an international perspective, which can more comprehensively discusses the logical relationship between digitalization and haze pollution. Second, the asymmetric impact of digitalization on haze pollution is further analyzed according to the level of economic development as well as environmental protection efforts in different countries, which further enriches the literature on haze pollution management. Third, this study presents a detailed theoretical analysis and empirical examination of the transmission mechanism of digitalization affecting haze pollution from the perspective of energy consumption intensity and energy consumption structure, which provides new ideas for haze pollution prevention and control. The remainder of the paper is structured as follows: Section 2 examines the influence mechanism and hypothesis. Section 3 describes methods and the data. Section 4 discusses the empirical results. Section 5 summarizes the conclusions and policy implications.

## 2. Mechanism Analysis and Hypothesis

### 2.1. The Direct Effects of Digitalization on Haze Pollution

The cross-regional properties of haze pollution and the quasi-public properties of environmental resources determine the complexity of haze pollution management issues [29]. The key to solving the problem of haze pollution control is to form a comprehensive environmental governance system involving government, businesses, and public participation with multi-party consultation and cooperation [30,31,32,33]. In the environmental governance system, the characteristics of dematerialization and virtualization enable digitalization to act as an interactive bridge between the government, the enterprise, and the public. In the construction of government environmental supervision models, sensors and data collectors are used to generate enviro-centric big data [34]. Data warehouses and monitoring tools are employed to strengthen the ecological information infrastructure of the central and local governments [35]. The internet environmental regulatory system provides the platform with real-time transmission and online interaction features for government departments to conduct environmental monitoring and governance. In the construction of a green production model in enterprises, asset specialization reduces the possibility of fixed assets being used for other purposes without losing production value. Traditional industries have difficulty obtaining funds for green production technology and innovation, so environmental pollution can be serious [36]. Digitalization makes knowledge and technology more modular and mobile to reduce asset specificity in traditional business activities [37] and promote the value arrangement of green creation and reduce haze pollution [38]. In the construction of public environmental supervision means, generally speaking, public participation in democratic decision-making and supervision not only represents the performance of political democracy but may contribute to effective, efficient, and legitimate decision-making [39,40]. In the digital era, social media such as Twitter and Facebook provide new channels and platforms for the public to participate in haze control, enriching the ways in which the public can participate in environmental governance [41]. The public can obtain ecological testing data, keep abreast of environmental conditions, and report corporate pollution through the internet online and other social monitoring platforms, effectively improving the effectiveness of environmental pollution management [42].

**Hypothesis** **1** **(H1).** *Digitalization can closely link the government, enterprises, and the public to form a closed loop for haze pollution prevention, which can effectively reduce haze pollution*.

### 2.2. The Indirect Effects of Digitalization on Haze Pollution

#### 2.2.1. Digitalization, Energy Consumption Intensity, and Haze Pollution

The virtual and permeable attributes of digitalization are highly compatible and applicable to the development goal of improving energy use efficiency and reducing energy consumption intensity. Specifically, digitalization can minimize energy consumption intensity in the following ways and thus curb haze pollution. At the macroscopic level, digitalization derived from cloud computing and the Internet of Things platform has a powerful network effect [43], which can organize and dispatch massive and scattered data resources across space and time. The powerful network effect breaks the information asymmetry in the social energy system [44]. Various types of energy are allocated to the links and fields with the highest utilization efficiency, optimizing the energy allocation between industries and enterprises, greatly improving energy use efficiency, and reducing energy consumption intensity. At the microcosmic level, with the support of the essential attributes of dematerialization and virtualization, digitalization can not only achieve the effective allocation of energy in the whole of society by reducing information asymmetry, but can deeply integrate with production technology and energy use technology, effectively promoting intelligent change of enterprise production processes and realizing the “lightness” of enterprise production methods [45,46]. Digitalization improves the coordination of production lines by empowering traditional production equipment with computing, communication, precision control, remote coordination, and self-management functions. As the digital level of the enterprise improves, enterprises can realize the organizational optimization of different processes on the basis of the dynamic production situations, reducing energy consumption and haze pollution [47].

**Hypothesis** **2** **(H2).** *Digitalization can play a suppressive role in haze pollution by reducing the energy consumption intensity of production processes*.

#### 2.2.2. Digitalization, Energy Consumption Structure, and Haze Pollution

From the perspective of energy consumption structure, it is important to increase the proportion of renewable energy consumption in energy consumption for haze pollution control. Digitalization can drive a green transformation of the energy consumption structure and thus reduce haze pollution by increasing the availability of renewable energy and the efficient allocation of renewable resources. First, digitalization can improve the availability of renewable energy. Compared with non-renewable energy sources, the power generated by renewable energy sources such as solar and wind energy is often unstable and difficult to collect and store [48]. Therefore, how to improve the accessibility and stability of renewable energy is crucial to optimizing the energy consumption structure. Taking solar power as an example, solar photovoltaic (PV) production mainly depends on radiant intensity, weather conditions, module performance, and the installation and operation of maximum power point tracking (MPPT) [49]. Among them, the MPPT, as the integration of digital and solar PV systems, continuously controls the direct current for the solar module, module string, or plant to generate maximum electrical power during the varying irradiation conditions and temperatures [50,51]. Second, digitalization enables efficient transportation and distribution of renewable resources. Compared to renewable energy production, efficient allocation and delivery of renewable resources are equally important [48]. Energy storage systems (ESSs), supported by digitalization, can help power companies meet consumers’ electricity demands stably and encourage renewable energy consumption by providing voltage support, smoothing their output fluctuations, and accurately balancing power flow in the network to match supply and demand [52].

**Hypothesis** **3** **(H3).** *Digitalization can optimize the energy consumption structure and reduce haze pollution by supporting renewable energy consumption*.

## 3. Methodology and Data

### 3.1. Methodology

#### 3.1.1. Benchmark Model

Traditional panel models such as mixed effects (OLS), fixed effects (FE), and random effects (RE) are prone to bias in the estimation of results due to endogeneity issues [53]. The system generalized method of moments (GMM) can effectively solve the serial autocorrelation and heteroskedasticity problems, thus significantly alleviating the endogeneity problem [54]. Therefore, this paper adopts the system GMM to verify the influence of digitalization on haze pollution. To test the plausibility of Hypothesis 1, a specific model was constructed as following:

(1) $$lnHP_{jt}=\alpha_{0}+\alpha_{1}lnHP_{j,t-1}+\alpha_{2}lnDIG_{jt}+\alpha_{3}lnGDP_{jt}+\alpha_{4}lnURBAN_{jt}+\alpha_{5}lnETI_{jt}+\alpha_{6}lnTF_{jt}+\varepsilon_{jt}\;$$

where the subscripts *j* and *t* represent country and year, respectively, and $lnHP_{jt}$ is the explanatory variable that indicates the level of haze pollution in country *j* in year *t*. Given that haze pollution is likely to have a time lag and the degree of haze pollution in the current period is expected to be related to the degree of haze pollution in previous periods, this study attaches the lagged period of the explanatory variable ($lnHP_{j,t-1}$) to Equation (1). $lnDIG_{jt}$ is the core independent variable denoting the level of digitalization in country *j* in year *t*. $lnGDP_{ji}$, $lnURBAN_{jt}$, $lnETI_{jt}$, and $lnTF_{jt}$ are control variables that present the level of economic development, urbanization, environmental technology innovation, and trade freedom in country *j* in year *t*, respectively. $\alpha_{0}$ and $\varepsilon_{jt}$ are the constant term and the random error term, respectively.

#### 3.1.2. Mediating Effect Model

To validate whether digitalization can inhibit haze pollution by reducing energy consumption intensity and optimizing the energy consumption structure, drawing on the research ideas of Baron and Kenny (1986), Mackinnon et al. (2007), and other scholars [55,56], this paper adopts a stepwise regression to test for the existence of a mediating effect. Stepwise regression covers three steps. In addition to Equation (1), the following two regressions were constructed.

(2) $$lnM_{jt}=\beta_{0}+\beta_{1}lnM_{j,t-1}+\beta_{2}lnDIG_{jt}+\beta_{3}lnGDP_{jt}+\beta_{4}lnURBNA_{jt}+\beta_{5}lnETI_{jt}+\beta_{6}lnTF_{jt}+\varepsilon_{jt}\;$$

(3) $$lnHP_{jt}=\delta_{0}+\delta_{1}lnHP_{j,t-1}+\delta_{2}lnDIG_{jt}+\delta_{3}lnM_{jt}+\delta_{4}lnGDP_{jt}+\delta_{5}lnURBAN_{jt}+\delta_{6}lnETI_{jt}+\delta_{7}lnTF_{jt}+\varepsilon_{jt}\;$$

First, Equation (1) is estimated to test whether haze pollution is affected by digitalization. Next, each of mediating variables, including energy consumption intensity and energy consumption structure, are regressed against digitalization as shown in Equation (2). Finally, haze pollution is regressed against both the main variable of digitalization, and the mediating variables in Equation (3), where $M_{j,t-1}$ is the mediating variable in Equation (2), which denotes energy consumption intensity ($EI_{jt}$) and energy consumption structure ($ES_{jt}$). Equation (2) also introduces a lag period of the intermediary variable ($M_{j,t-1}$) to reduce the possibility of missing variables and ensure the robustness of the model set. Other variables in Equation (2) have the same meaning as in Equation (1). The definition of the variables in Equation (3) is the same as in Equations (1) and (2). If $\beta_{2}$ in Equation (2), $\delta_{2}$ and $\delta_{3}$ in Equation (3) are significant, then $M_{j,t-1}$ has a partial mediating effect. In contrast, if $\beta_{2}$ in Equation (2) and $\delta_{3}$ in Equation (3) are significant, and $\delta_{2}$ in Equation (3) is not significant, $M_{j,t-1}$ has a complete mediating effect.

### 3.2. Variables

#### 3.2.1. Explained Variable

Haze pollution (HP) is the explained variable. The main components of haze pollution include ${\mathrm{SO}}_{2}$, ${\mathrm{NO}}_{x}$, and respirable particles of ${\mathrm{PM}}_{2.5}{\;\mathrm{and}\;\mathrm{PM}}_{10}$. Compared with ${\mathrm{PM}}_{10}$ and other particles, ${\mathrm{PM}}_{2.5}$ is smaller in diameter, less likely to settle, and can be monitored using remote sensing technology, which can better quantify haze pollution and reflect the true level of haze pollution [57,58,59]. Consequently, the annual average ${\mathrm{PM}}_{2.5}$ concentration of each country was chosen to characterize the degree of haze pollution in this paper.

#### 3.2.2. Core Explanatory Variable

Digitalization (DIG) is the core explanatory variable. In the era of the sharing economy, digitalization is sweeping across the world. Combining digitalization with traditional production methods generates new opportunities for haze pollution prevention and control through innovative energy management, smart factories, smart cities, and smart homes. Drawing on the findings of Ramos et al. (2021) [60], this article uses the internet penetration rate (NET) to measure the level of digitalization. In general, the higher the internet penetration rate, the higher the level of digitalization. In addition, this paper adopts mobile phone penetration rate (MOB) as a proxy variable for internet penetration rate to conduct robustness tests.

#### 3.2.3. Mediating Variables

Energy consumption intensity (EI) and energy consumption structure (ES) are mediating variables. Energy consumption intensity is expressed as energy consumption per unit of GDP. Therefore, the relative lower energy intensity indicates that the comprehensive efficiency of energy utilization, which is conductive to improving the quality of environment [58]. The energy consumption structure is the share of renewable energy in the final energy consumption. The optimization of energy consumption structure means an increase in renewable energy consumption, which can effectively reduce haze pollution [61].

#### 3.2.4. Control Variables

Aside from digitalization and energy consumption, considering the possible contribution of other factors to haze pollution, we selected a series of other factors at the national level as control variables, which can be broadly classified into two categories: economy and environment. The economic factors mainly include the level of economic development (GDP), the level of urbanization (URBAN), and the freedom of trade (TF) [58,62,63]. Specifically, economic growth significantly improves the degree of environmental awareness and responsibility of economic entities [64]. In general, countries with a high level of economic development are more concerned about haze pollution issues. In the process of urbanization, a large number of people are concentrated in cities, resulting in huge energy consumption and serious haze pollution [65]. Worldwide trade expansion is often accompanied by an increase in production activities and higher energy consumption. Thus, this expansion trend has a significant negative impact on environmental [66]. For the environmental factor, the proportion of environmental protection technology patents to the total number of patent applications was selected to represent the degree of environmental technology innovation (ETI) [67]. Environmental technology innovation can improve resource use efficiency, but its marginal role is diminishing, and a rapidly increasing economic scale may still require more investment in natural resources [68].

### 3.3. Data

Based on data availability, we obtained cross-country panel data for 81 countries from 2010–2019 after excluding samples with a large number of missing values. The data for ${\mathrm{PM}}_{2.5}$ and the degree of environmental technology innovation were obtained from the OECD database. The data for internet penetration rate, cell phone penetration rate, energy consumption intensity, energy consumption structure, GDP, and urbanization levels were retrieved from the World Bank database, and the freedom of trade came from the Fraser Institute in Canada. All the sample countries are listed in Table 1. The descriptive statistics of each variable are shown in Table 2. According to the definition of developed and developing countries by the United Nations Conference on Trade and Development (UNCTAD) and World Bank concerning determination of whether to implement a carbon tax, the 81 countries have been classified into two subsamples.

## 4. Empirical Results and Discussion

### 4.1. Analysis of Direct Effect

#### 4.1.1. Result of Benchmark Regression

The system GMM method was used to estimate the parameters of the constructed dynamic panel model of Equation (1). The estimation results are shown in Table 3. The explanatory variables in columns (1)–(3) are internet penetration rate, and the explanatory variables in columns (4)–(6) are mobile phone penetration rate. As can be seen from the estimation results in column (1), the AR(1) is less than 0.05, and AR(2) is greater than 0.1, which implies there is no endogeneity problem in the model. Hansen test results cannot reject the null hypothesis that the instrumental variables are over-identified at the 0.1 significant level, indicating that the variables selected in this paper are effective. According to the research of Bond (2002), to confirm the validity of the system GMM estimation results, this study employs the OLS and FE model to estimate the dynamic panel model once more [69]. As shown in columns (1) to (3), the estimated coefficient of the lagged explanatory variables in the system GMM is between the FE estimation result and the OLS estimation result, which indicates that the system GMM estimation result is valid.

In column (1), the impact coefficient of haze pollution with one-period lag is significantly positive, demonstrating that haze pollution is cumulative and persistent, which further justifies the construction of a dynamic panel model for analysis is necessary. The estimated coefficient of digitalization is −0.0162 and passes the 0.01 significance level, which correlates negatively with haze pollution. For every 1% increase in digitalization, haze pollution will be reduced by 0.0612 percentage points. On the one hand, digitalization captures and predicts changes in supply and demand in the energy market on time through data collection and transmission, calculation, and analysis. This greatly improves energy consumption efficiency and intensity, reducing haze pollution. On the other hand, by empowering traditional production factors such as land, labor, and capital, digitalization promotes profound changes in conventional production and lifestyles, and accelerates the innovation of production equipment and green technologies. This ultimately achieves green development and reduces haze pollution. Hypothesis 1 is correct. Our results also confirm the research results of Yan et al. (2021) [70].

The estimated coefficient of the economic development level is −0.0015 and statistically significant, showing that economic growth is a crucial tactic for the pursuit of haze pollution control. This outcome found support from the previous study [71]. Urbanization shows a positively significant impact on haze pollution, which is in line with the research of Liu et al. (2020) [72]. When the population is concentrated, non-renewable resources are heavily used, and haze pollution increases in the process of urbanization. The estimated coefficient of environmental technology innovation is significantly positive, reflecting that the environmental effects of environmental technology innovation are not significant at this stage. Conversely, the influx of capital in the environmental sector has increased energy consumption for environmental activities and thus increased haze pollution. The result sharply contrasts with Yi et al. (2021), who found that technological innovation can significantly reduce the ${\mathrm{PM}}_{2.5}$ concentration [73]. Trade freedom significantly affects haze pollution with coefficients of −0.0693, suggesting that the technological spillovers of foreign trade activities lead to green technological advancements and management approaches coordinated with energy-saving measures. This finding is consistent with the result of Xu et al. (2019) [74]. As can be seen from columns (4) to (6), the estimated results of each variable are compatible with the calculated results of columns (1) to (3) when mobile phone penetration is seen as the explanatory variable, and the model is robust.

#### 4.1.2. Heterogeneity Analysis

(1) Regression analysis of different Economic development levels

The UNCTAD database divides countries into developed and developing countries in terms of economic development level. Accordingly, we separated the total sample into two sub-samples, developed and developing countries, for the empirical study to explore the heterogeneity of the impact of digitalization on haze pollution under different levels of economic development. The results are shown in Table 4, which show that digitalization, represented by the internet penetration rate, significantly inhibits haze pollution in both developed and developing countries. However, the absolute value of the estimated coefficient of digitalization is greater in developed countries than in developing countries, suggesting that the suppressive effect of digitalization on haze pollution is more pronounced in developed countries. The reason for this may be that developed countries have strong economies, dynamic markets, and a high degree of economic openness, which provide fertile ground for the sprouting and growth of digitalization. Rapidly developing digitalization integrates deeply with all industries, making the supply side effectively connect to the demand side, and energy supply accurately matches energy demand, which minimizes haze pollution. Compared with developed countries, the suppression effect of digitalization on haze pollution in developing countries lacks strong support due to the low level of economic development and inadequate digital infrastructure. It is an essential issue for developing countries to consider how to take the free ride of digitalization to develop emerging industries, transform traditional industries, and effectively control haze pollution. The estimation results with mobile phone penetration rate as the explanatory variable further confirm the robustness of this conclusion.

(2) Regression analysis of different environmental protection efforts

As a traditional tool to address environmental issues, whether or not to implement a carbon tax can measure how a country attaches importance to environmental protection. Therefore, the implementation of a carbon tax was taken as the evaluation standard for environmental protection efforts. Referring to the State and Trends of Carbon Pricing 2022 report published by the World Bank, we divided the sample into two categories: implemented carbon tax countries and unimplemented carbon tax countries. The results are shown in Table 5. In columns (1) and (3), the estimated coefficients of digitalization are significantly negative, implying that digitalization has a dampening influence on haze pollution regardless of whether a carbon tax is implemented. From the perspective of the absolute value of the coefficient, compared with countries without a carbon tax, the suppression effect of digitalization on haze pollution is more obvious in countries that implement a carbon tax. This conclusion further proves the correctness of Porter’s hypothesis (1995) [32].

The Porter hypothesis suggests that environmental protection policies, such as carbon taxes, have dual economic and environmental benefits. The carbon tax pressures companies to innovate and progress in green technology. Under this pressure, companies further enhance investment in research and development to achieve technological innovation and effectively avoid haze pollution. Consequently, companies in countries implementing a carbon tax are more motivated to carry out digital transformation, improve energy efficiency, and achieve green development. On the contrary, under the assumption of rational economics, it is difficult for enterprises to internalize the external environmental cost generated in the production process, so digitalization cannot effectively exert the inhibitory effect on haze pollution in countries with no implemented a carbon tax. The results remain largely unchanged when mobile phone penetration rate is used as the explanatory variable.

### 4.2. The Analysis of Indirect Effect

#### 4.2.1. The Mediating Effect of Energy Consumption Intensity

The aforementioned theoretical analysis shows that energy consumption intensity is an important channel for a country’s digital development to reduce haze pollution. In order to verify the validity of this hypothesis, we took energy consumption intensity as a mediating variable and perform a stepwise regression analysis according to the previous Equations (1)–(3). The estimated results are shown in Table 6. In Table 6, column (1) corresponds to Equation (1), which is the reported result in column (1) of Table 3. The estimated coefficient of digitalization is significantly negative, showing that the total negative effect of digitalization on haze pollution is significant. Column (2) corresponds to Equation (2). The estimated coefficient of digitalization is −0.1410 and passes the significance test at the 0.01 level, illustrating that digitalization can effectively reduce energy consumption intensity. Column (3) corresponds to Equation (3). The effects of digitalization on energy consumption intensity and haze pollution are both significant, and the influence coefficients are −0.0109 and 0.0165, respectively, which means energy consumption intensity has played an effective intermediary role in digitalization and haze pollution. Combined with the estimation results in column (1), the absolute value of the digitalization coefficient reduces after adding the mediating variables, indicating that energy consumption intensity plays a partially mediating role. This further shows that energy consumption intensity is a channel for digitally suppressing haze pollution, and the mediating effect accounts for 3.80% of the total impact. The assumption of Hypothesis 2 is correct. The results with cell phone penetration as the explanatory variable remain largely unchanged.

#### 4.2.2. The Mediating Effect of Energy Consumption Structure

Digitalization can obviously translate into green productivity, not only to reduce unnecessary consumption of energy but also to boost clean energy production and energy consumption structure transformation. Based on the previous theoretical analysis, we further examined the mechanism of digital development on haze pollution by considering energy consumption structure as a mediating variable. The estimated results are shown in Table 7. In Table 7, columns (1)-(3) correspond to Equations (1)-(3) separately. The results in column (1) are the same as those reported in column (1) of Table 6. In column (2), digitalization is positive and significant, indicating that digitalization has a significant positive correlation with energy consumption structure. This result is as expected. Column (3) shows that the impact of digitalization and energy consumption structure on haze pollution is negative, implying that energy consumption structure plays an effective intermediary channel in digitalization and haze pollution, and the mediating effect accounts for 4.33% of the total effect. Hypothesis 3 is verified. After re-estimating the model with mobile phone penetration rate as a proxy variable of digitalization, it was found that the energy consumption structure still plays a partial mediating effect between digitalization and haze pollution, which further proves the rationality of Hypothesis 3.

## 5. Conclusions

Digitalization is the foundation of a new round of technology and industry revolutions. By accelerating the development of digitalization to promote energy consumption conservation and cleanliness is not only the key to effective management of haze pollution but also essential to improve residents’ physical health. Based on cross-country panel data for 81 countries from 2010–2019, this paper empirically examines the impact and mechanism of digitalization on haze pollution from energy consumption intensity and energy consumption structure using systematic GMM and mediating effect models. The empirical results show that digitalization can effectively suppress haze pollution. Further heterogeneity analysis shows that the inhibitory effect of digitalization on haze pollution is more evident in developed countries and countries implementing carbon taxes. The results of the mediation effect model show that digitalization can also indirectly suppress haze pollution by reducing energy consumption intensity and optimizing energy consumption structure.

We propose the following policy recommendations based on the above research findings.

First, based on the haze pollution control framework, government departments should make overall plans for the digital industry and industrial digital development pattern and give full play to the positive role of digitalization in haze pollution control by constructing a digital development system with coherent functions, clear division of labor, and transparent supervision. Furthermore, the government should focus on accelerating the construction of digital facilities such as cloud computing, IPV6 address numbers, and big data, expanding the scale of digital industries, providing support for promoting digital transformation and traditional industry upgrades, and thereby reducing the use of conventional non-renewable energy sources, improving energy consumption efficiency, and reducing haze pollution.

Second, considering the heterogeneity of the impact of digitalization on haze pollution, countries should be able to implement digital strategies according to local conditions. For developing countries, the focus may be on building digital infrastructure and strengthening the application and popularization of information technology, with the internet as the core, to broader the coverage of digital infrastructure and improve the digital haze pollution management capability. Countries that have not implemented a carbon tax may learn from the digital development policies and carbon tax policies of countries that have implemented a carbon tax, and formulate a scientific and reasonable carbon tax policy system, according to their economic development stage, to further strengthen the environmental effects of digitalization.

Third, the energy consumption intensity and the energy consumption structure effect caused by digitalization are the two crucial driving forces in reducing haze pollution. Therefore, when formulating haze pollution control policies, the government needs to further clarify the coordination between energy conservation policies and haze control policies to provide effective policy protection for haze pollution control. In addition, the government should use digital technologies such as big data, cloud computing, and the internet of things to build a digital energy information platform to allocate energy across time and regions, promote energy efficiency, reduce haze pollution, and achieve the goal of improving health level and life quality.

## Figures and Tables

**Table 1:** List of sample countries in this research.

Economic Development Level				Whether to Implement a Carbon Tax			
Developed Countries		Developing Countries		Carbon Tax Implemented		Carbon Tax Not Implemented	
Australia	Poland	Mexico	Algeria	Argentina	United States	Australia	Belgium
Austria	Portugal	Argentina	Armenia	Mexico	Columbia	Canada	Czech
Belgium	Slovakia	Azerbaijan	Bangladesh	Brazil	Iceland	Greece	Hungary
Canada	Spain	Brazil	China	Sweden	Finland	Italy	Slovakia
Czech	Sweden	Columbia	Costa Rica	Norway	Denmark	Albania	Algeria
Denmark	Switzerland	Dominican	Ecuador	Germany	Ireland	Armenia	Azerbaijan
Bosnia and Herzegovina	United Kingdom	United Arab Emirates	Ethiopia	United Kingdom	Netherlands	Bosnia and Herzegovina	United Arab Emirates
Finland	United States	Georgia	Ghana	Luxembourg	Spain	Bangladesh	Bulgaria
Germany	Albania	India	Indonesia	Portugal	France	Costa Rica	Croatia
Greece	Belarus	Iran	Jordan	Switzerland	Slovenia	Cyprus	Dominican
Hungary	France	Kazakhstan	Kenya	Austria	Serbia	Ecuador	Egypt
Iceland	Bulgaria	Malaysia	Morocco	Ukraine	Poland	Ethiopia	Georgia
Ireland	Croatia	Nigeria	Pakistan	Latvia	Estonia	Ghana	India
Italy	Cyprus	Panama	Peru	Kazakhstan	Pakistan	Iran	Israel
Japan	Estonia	Qatar	South Africa	China	Thailand	Jordan	Kenya
Korea, Republic of	Israel	Thailand	Tunisia	Indonesia	New Zealand	Lithuania	Malaysia
Luxembourg	Latvia	Egypt	Uruguay	Vietnam	Korea, Republic of	Malta	Mongolia
Netherlands	Lithuania	Vietnam	Singapore	Japan	South Africa	Morocco	Nigeria
New Zealand	Malta	Mongolia				Panama	Qatar
Norway	Romania					Peru	Romania
Russia	Slovenia					Russia	Singapore
Ukraine	Serbia					Tunisia	Belarus
						Uruguay	

**Table 2:** Descriptive statistics of variables.

Variables	**Obs**	**Mean**	**Std. Dev**	**Min**	**Max**
lnHP	810	2.9566	0.6415	1.6734	4.5627
lnNET	810	4.0094	0.6248	−0.2877	4.6022
lnMOB	810	4.7435	0.2713	2.0567	5.3596
lnES	810	2.5977	1.2479	−2.8302	4.5445
lnEI	810	1.3940	0.4244	0.2776	2.7587
lnGDP	810	7.5992	1.7747	1.5810	12.2750
lnURBAN	810	4.1987	0.3097	2.8519	4.6052
lnETI	810	2.4827	0.5130	−0.1744	4.5282
lnTF	810	2.0049	0.1843	0.9361	2.2575

**Table 3:** The result of benchmark regression.

Variables	System GMM (1)	FE (2)	OLS (3)	System GMM (4)	FE (5)	OLS (6)
L.lnHP	0.9770 *** (0.0026)	0.5846 *** (0.0261)	0.9988 *** (0.0043)	0.9827 *** (0.0033)	0.5835 *** (0.0266)	1.0036 *** (0.0043)
lnNET	−0.0612 *** (0.0012)	−0.0302 ** (0.0128)	−0.0226 *** (0.0065)			
lnMOB				−0.1767 *** (0.0052)	0.0207 (0.0238)	−0.0213 ** (0.0106)
lnGDP	−0.0015 ** (0.0006)	0.0483 *** (0.0171)	−0.0001 (0.0012)	−0.0013 (0.0012)	0.0441 ** (0.0172)	−0.0004 (0.0012)
lnURBAN	0.0826 *** (0.0045)	−0.4850 *** (0.1540)	0.0254 ** (0.0104)	0.0588 *** (0.0047)	−0.7774 *** (0.1271)	0.0112 (0.0098)
lnETI	0.0043 *** (0.0004)	0.0027 (0.0046)	0.0034 (0.0042)	0.0068 *** (0.0004)	0.0038 (0.0045)	0.0026 (0.0044)
lnTF	−0.0693 *** (0.0079)	−0.0896 (0.0298)	−0.0099 (0.0148)	−0.1037 *** (0.0114)	−0.0870 *** (0.0300)	−0.0193 (0.0141)
CONS	0.0933 *** (0.0323)	3.1815 *** (0.6110)	0.0051 (0.0527)	0.8332 *** (0.0546)	4.2162 *** (0.4962)	0.0646 (0.0582)
AR(1)	0.0000			0.0000		
AR(2)	0.1160			0.1000		
Hansen−Test	0.9810			0.9850		
$R^{2}$		0.8940	0.9926		0.8413	0.9916

Note: “***” and “**” mean significance at the level of 0.01 and 0.05 respectively; standard errors in parentheses.

**Table 4:** Results of the heterogeneity test based on the economic development level.

Variables	Developed Country		Developing Country	
	(1)	(2)	(3)	(4)
L.lnHP	0.3973 *** (0.0097)	0.3062 *** (0.0424)	0.7498 *** (0.0202)	0.5929 *** (0.0558)
lnNET	−0.2499 *** (0.0171)		−0.0255 *** (0.0069)	
lnMOB		−0.9426 *** (0.1766)		−0.1228 * (0.0644)
lnGDP	0.0841 *** (0.0062)	0.0308 (0.0330)	−0.1104 *** (0.0127)	−0.1552 *** (0.0150)
lnURBAN	−0.9325 *** (0.0922)	−1.3785 (0.4100)	−0.1627 * (0.0823)	−0.3268 (0.2117)
lnETI	0.0152 *** (0.0031)	0.0502 *** (0.0080)	−0.0083 (0.0060)	−0.0459 *** (0.0063)
lnTF	−0.5689 *** (0.0423)	−0.9322 *** (0.1784)	−0.5305 *** (0.0517)	−0.7500 *** (0.2052)
CONS	7.1203 *** (0.3639)	13.7963 *** (1.9702)	3.4512 *** (0.2521)	5.9722 *** (0.8921)
AR(1)	0.0000	0.0000	0.0000	0.0140
AR(2)	0.5720	0.3260	0.1360	0.1160
Hansen−Test	0.2940	0.1140	0.6580	0.2230

Note: “***”and “*” mean significance at the level of 0.01 and 0.05 respectively; standard errors in parentheses.

**Table 5:** Results of the heterogeneity test based on environmental effort.

Variables	Implemented Carbon Tax		Unimplemented Carbon Tax	
	(1)	(2)	(3)	(4)
L.lnHP	0.6208 *** (0.0236)	0.1847 *** (0.0319)	0.9335 *** (0.0198)	0.9490 *** (0.0097)
lnNET	−0.1062 *** (0.0213)		−0.0381 *** (0.0066)	
lnMOB		−0.1406 ** (0.0568)		−0.0772 *** (0.0138)
lnGDP	0.0933 *** (0.0113)	0.2277 *** (0.0219)	−0.0855 *** (0.0104)	−0.0082 *** (0.0029)
lnURBAN	−0.0856 (0.0583)	−0.8720 *** (0.1714)	0.1309 *** (0.0221)	−0.0617 * (0.0360)
lnETI	−0.0511 *** (0.0076)	0.0549 *** (0.0098)	0.0498 *** (0.0029)	−0.0059 *** (0.0014)
lnTF	−0.9299 *** (0.1428)	−1.2398 *** (0.1418)	−0.2666 *** (0.0489)	0.0128 (0.0371)
CONS	3.0299 *** (0.4326)	7.0395 (0.4809)	0.8093 *** (0.1475)	0.8192 *** (0.1475)
AR(1)	0.0000	0.0000	0.0000	0.0000
AR(2)	0.2560	0.2460	0.5900	0.2880
Hansen−Test	0.6440	0.3450	0.2550	0.2550

Note: “***”, “**”, and “*” mean significance at the level of 0.01, 0.05, and 0.1, respectively; standard errors in parentheses.

**Table 6:** Mechanism test results of energy consumption intensity as the mediating variable.

Variables	lnHP	lnEI	lnHP	lnHP	lnEI	lnHP
	(1)	(2)	(3)	(4)	(5)	(6)
L.lnHP	0.9770 *** (0.0026)		1.0025 *** (0.0009)	0.9827 *** (0.0033)		0.9678 *** (0.0022)
L.lnEI		0.3306 *** (0.0729)			0.8643 *** (0.0068)	
ln NET	−0.0612 *** (0.0012)	−0.1410 *** (0.0320)	−0.0109 *** (0.0019)			
lnMOB				−0.1767 *** (0.0052)	−0.0186 ** (0.0089)	−0.0725 *** (0.0040)
lnEI			0.0165 *** (0.0027)			0.0136 *** (0.0035)
lnGDP	−0.0015 ** (0.0006)	−0.0039 (0.0114)	−0.0062 *** (0.0009)	−0.0013 (0.001)	−0.0242 *** (0.0028)	−0.0009 (0.0009)
lnURBAN	0.0826 *** (0.0045)	0.4499 ** (0.2164)	0.0125 (0.0076)	0.0588 *** (0.0047)	0.0705 *** (0.0121)	0.0060 (0.0043)
lnETI	0.0043 *** (0.0004)	0.0426 (0.0287)	0.0043 ** (0.0018)	0.0068 *** (0.0004)	−0.0009 (0.0015)	0.0026 ** (0.0011)
lnTF	−0.0693 *** (0.0079)	−0.8045 *** (0.1845)	0.0513 *** (0.0066)	−0.1037 *** (0.0114)	−0.2036 *** (0.0142)	−0.0438 *** (0.0035)
CONS	0.0933 *** (0.0323)	1.1422 * (0.6245)	−0.1194 *** (0.0189)	0.8332 *** (0.0546)	0.5587 *** (0.0470)	0.4699 *** (0.0259)
AR(1)	0.0000	0.0380	0.0000	0.0000	0.0000	0.0000
AR(2)	0.1160	0.1830	0.1070	0.1000	0.4660	0.1040
Hansen−Test	0.9810	0.1280	0.9090	0.9850	0.1680	0.2710

Note: “***”, “**”, and “*” mean significance at the level of 0.01, 0.05, and 0.1, respectively; standard errors in parentheses.

**Table 7:** Mechanism test results of energy consumption structure as the mediating variable.

Variables	lnHP	lnES	lnHP	lnHP	lnES	lnHP
	(1)	(2)	(3)	(4)	(5)	(6)
L.lnHP	0.9770 *** (0.0026)		0.9061 *** (0.0120)	0.9827 *** (0.0033)		0.9755 *** (0.0041)
L.lnES		0.9472 *** (0.0013)			0.8579 *** (0.0104)	
lnNET	−0.0612 *** (0.0012)	0.1000 *** (0.0029)	−0.0887 *** (0.0074)			
lnMOB				−0.1767 *** (0.0052)	0.2599 *** (0.0444)	−0.1165 *** (0.0058)
lnES			−0.0265 *** (0.0070)			−0.0110 *** (0.0013)
lnGDP	−0.0015 ** (0.0006)	−0.0108 *** (0.0014)	0.0028 (0.0086)	−0.0013 (0.0012)	0.0233 *** (0.0064)	−0.0095 *** (0.0017)
lnURBAN	0.0826 *** (0.0045)	−0.1172 *** (0.0079)	0.1084 *** (0.0391)	0.0588 *** (0.0047)	−0.1369 * (0.0708)	0.0325 *** (0.0085)
lnETI	0.0043 *** (0.0004)	0.0224 *** (0.0017)	−0.0227 *** (0.0034)	0.0068 *** (0.0004)	0.0843 *** (0.0058)	−0.0043 *** (0.0013)
lnTF	−0.0693 *** (0.0079)	0.1737 *** (0.0100)	−0.2493 *** (0.0522)	−0.1037 *** (0.0114)	0.39145 *** (0.0481)	−0.1086 *** (0.0091)
CONS	0.0933 *** (0.0323)	−0.0742 ** (0.0298)	0.7666 *** (0.1940)	0.8332 *** (0.0546)	−1.4322 *** (0.3734)	0.8045 *** (0.0526)
AR(1)	0.0000	0.0070	0.0000	0.0000	0.0030	0.0000
AR(2)	0.1160	0.1660	0.1160	0.1000	0.6180	0.1350
Hansen−Test	0.9810	0.9930	0.1980	0.9850	0.5410	0.9940

Note: “***”, “**”, and “*” mean significance at the level of 0.01, 0.05, and 0.1, respectively; standard errors in parentheses.

## Data Availability

The data in the manuscript come the OECD database, the World Bank database, and the Fraser Institute in Canada.
